# Quantification of Epicardial Adipose Tissue Volume and Attenuation for Cardiac CT Scans Using Deep Learning in a Single Multi-Task Framework

**DOI:** 10.31083/j.rcm2312412

**Published:** 2022-12-20

**Authors:** Musa Abdulkareem, Mark S. Brahier, Fengwei Zou, Elisa Rauseo, Ijeoma Uchegbu, Alexandra Taylor, Athanasios Thomaides, Peter J. Bergquist, Monvadi B. Srichai, Aaron M. Lee, Jose D. Vargas, Steffen E Petersen

**Affiliations:** ^1^Barts Heart Centre, Barts Health National Health Service (NHS) Trust, EC1A 4NP London, UK; ^2^National Institute for Health Research (NIHR) Barts Biomedical Research Centre, William Harvey Research Institute, Queen Mary University of London, E1 4NS London, UK; ^3^Health Data Research UK, NW1 2BE London, UK; ^4^Georgetown University School of Medicine, Washington, DC 20007, USA; ^5^Duke University Hospital, Durham, North Carolina, NC 27710, USA; ^6^Montefiore Medical Centre, Bronx, NY 10467, USA; ^7^Northeastern University, Boston, MA 02115, USA; ^8^MedStar Heart and Vascular Institute, Washington, DC 20010, USA; ^9^Veterans Affairs Medical Center, Washington, DC 20422, USA; ^10^The Alan Turing Institute, NW1 2BE London, UK

**Keywords:** deep learning, CT, epicardial adipose tissue, EAT, volume, attenuation, density

## Abstract

**Background:**

Recent studies have shown that epicardial adipose tissue (EAT) is an independent atrial fibrillation (AF) prognostic marker and has influence on the myocardial function. In computed tomography (CT), EAT volume (EATv) and density (EATd) are parameters that are often used to quantify EAT. While increased EATv has been found to correlate with the prevalence and the recurrence of AF after ablation therapy, higher EATd correlates with inflammation due to arrest of lipid maturation and with high risk of plaque presence and plaque progression. Automation of the quantification task diminishes the variability in readings introduced by different observers in manual quantification and results in high reproducibility of studies and less time-consuming analysis. Our objective is to develop a fully automated quantification of EATv and EATd using a deep learning (DL) framework.

**Methods:**

We proposed a framework that consists of image classification and segmentation DL models and performs the task of selecting images with EAT from all the CT images acquired for a patient, and the task of segmenting the EAT from the output images of the preceding task. EATv and EATd are estimated using the segmentation masks to define the region of interest. For our experiments, a 300-patient dataset was divided into two subsets, each consisting of 150 patients: Dataset 1 (41,979 CT slices) for training the DL models, and Dataset 2 (36,428 CT slices) for evaluating the quantification of EATv and EATd.

**Results:**

The classification model achieved accuracies of 98% for precision, recall and $F_{1}$ scores, and the segmentation model achieved accuracies in terms of mean (± std.) and median dice similarity coefficient scores of 0.844 (± 0.19) and 0.84, respectively. Using the evaluation set (Dataset 2), our approach resulted in a Pearson correlation coefficient of 0.971 ($R^{2}$ = 0.943) between the label and predicted EATv, and the correlation coefficient of 0.972 ($R^{2}$ = 0.945) between the label and predicted EATd.

**Conclusions:**

We proposed a framework that provides a fast and robust strategy for accurate EAT segmentation, and volume (EATv) and attenuation (EATd) quantification tasks. The framework will be useful to clinicians and other practitioners for carrying out reproducible EAT quantification at patient level or for large cohorts and high-throughput projects.

## 1. Introduction 

### 1.1 Background

Epicardial adipose tissue (EAT), the fat located between the myocardium and the visceral pericardium [1] which serves as an energy store [2], has been hypothesized as a contributor to the inflammatory burden via paracrine mechanisms [3]. EAT has been suggested as an independent marker for cardiovascular risk [2, 4, 5, 6, 7, 8] and, in particular, as an independent atrial fibrillation (AF) prognostic marker [9]. In computed tomography (CT), EAT volume (EATv) and density (EATd) are parameters that are often used to quantify EAT [8]. EATv refers to the extent of EAT accumulation and increased EATv has been found to correlate with the prevalence and the recurrence of AF after ablation therapy [1, 10, 11, 12, 13, 14, 15]. In addition to AF, increased EATv is also associated with atherosclerosis [16, 17], carotid stiffness [18], myocardial infarction [19], and coronary artery calcification [20, 21]. EATv has been associated with severity of coronary artery disease (CAD) [6, 22, 23]. A higher EATd (i.e., radiodensity of EAT) in CT images is correlated with inflammation as a result of arrest of lipid maturation [24]. Researchers have also suggested a link between EATd and high risk mortality and plaque presence and progression [24].

These health risk factors emphasize the need for direct quantification of EAT (i.e., EATv and EATd). However, manual quantification is time-consuming to accomplish in clinical practice in the light of the high workload on physicians and radiographers, and so EAT is not routinely quantified. Automation of the quantification task diminishes the variability in readings introduced by different observers and removes the high dependence of state-of-the-art methods on user interaction for EAT segmentation, resulting in high reproducibility of studies and less time-consuming analysis. In general, fully automated quantification of EATv and EATd requires advanced techniques and, in this work, we propose a deep learning (DL) framework for carrying out the estimation of these two quantities autonomously. DL techniques, a set of machine learning methods, have proved to be very effective for automated detection and segmentation of a wide range of medical images with a high degree of accuracy [25, 26, 27, 28, 29]. The proposed methodology, therefore, mainly consists of image classification and segmentation DL-based models to perform the desired quantification. We compare the performance of our approach to other DL approaches proposed in the literature for accomplishing EAT quantification.

### 1.2 Related Work

The segmentation of EAT in cardiac CT (CCT) image slices is important for EAT quantification and various semi-automatic segmentation approaches have been developed [30, 31, 32]. An overview of these approaches can be described as follows: after initial preprocessing involving the removal of all other structures in the CT images apart from the heart, these methods require an expert to scroll through the CT slices to identify some control points along the border of the pericardium, then use some interpolation methods (such as the cubic spline function techniques) to obtain smooth pericardial contour, and then identify the pericardial fat by thresholding.

Fully automated and semi-automated non-DL based EAT segmentation approaches have also been developed [33, 34, 35, 36, 37, 38]. The time taken for obtaining the segmentation masks per patient, according to [35], could be more than 15 minutes for such fully automated approaches. The DL-based automatic EAT quantification methods that have been reported for EAT segmentation or quantification include those by Commandeur *et al*. [39] and Li *et al*. [40]. In Commandeur *et al*. [39], two DL-based models were developed; one is used to determine heart limits and perform heart segmentation (i.e., thoracic mask segmentation) and the other was used in combination with a statistical shape model for the detection of the pericardium. EAT was then quantified by further post-processing using thresholding [–190, –30] HU. In Li *et al*. [40], a DL-based model was developed for the segmentation of the pericardium across multiple adjacent slices using multiple slices as input to the model. A smoothing operation is then employed by finding a solution to a partial differential equation of the 3-dimensional gradient vector flow in order to reduce the prediction of false positive and negative regions in the segmented pericardial images. EAT is then deduced by thresholding [–175, –15] HU.

To our knowledge, there is no unified method in the literature capable of both autonomous EATv and EATd. Methods exist for the EAT segmentation and EATv estimation [39, 40, 41, 42], but our approach differs from these methods in that EAT segmentation does not require any further post-processing (e.g., thresholding or smoothening operations with filters) after the prediction with the DL-based EAT segmentation model. Also, no previous work has attempted to estimate EATd alone with DL nor combine the estimation of EATv and EATd. Although some authors, such as [41, 42], have stated their quantification of EATd as the mean attenuation of EAT segmented using DL models, we are not aware of any previous work that demonstrates or carried out practical analysis of the EATd quantification using DL. Our results in the analysis of EATd strengthens the correctness of our approach of EAT segmentation and emphasizes the correctness of the results obtained for EATv estimation.

## 2. Materials and Methods

**Fig. 1.**
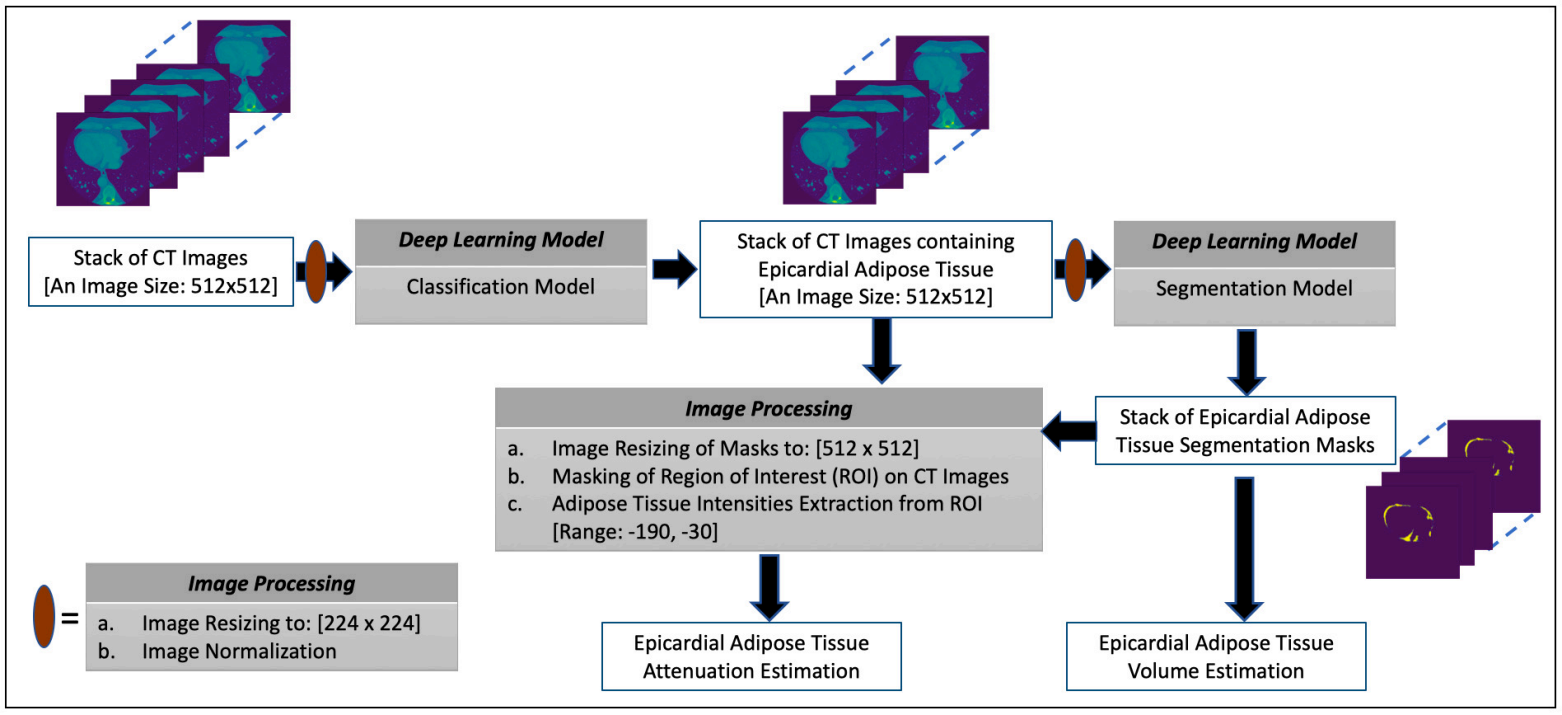
**The overview of the proposed framework for estimating the volume and attenuation of EAT**.

In this paper, the presentations related to DL follow the recommendation of the Proposed Requirements for Cardiovascular Imaging-Related Machine Learning Evaluation guidance [43]. The overview of the proposed framework for estimating the volume and attenuation of EAT is shown in Fig. 1 and consists of two DL models. The classification model performs the task of selecting images containing the EAT from the set of all CT images acquired for a given patient. The image segmentation model obtains the segmentation masks marking the regions of the EAT from the selected images of the preceding process. The estimation of EATv is computed using the segmentation masks while the mean attenuation of the totality of EATv, that is EATd (i.e., mean density of EAT) [8], is quantified by extracting the intensity values of the EAT from the CT images using the masks to define the region of interest (ROI). More details on these processes are given in subsequent subsections.

### 2.1 Data Acquisition and Analysis Tools

#### 2.1.1 Study Population

We included 300 patients for this retrospective observational study who underwent catheter ablation for symptomatic, anti-arrhythmic medication-refractory atrial fibrillation at MedStar Georgetown University Hospital in Washington, D.C. All patients gave written informed consent and underwent CCT for pre-operative assessment. The de-identification of the images was carried out prior to analysis. The approval for the study was given by the Georgetown University Institutional Review Board (STUDY-0400, approved 7/20/2017). A total of 78,407 images from the 300 patients were available for the study. This dataset is used for all the analysis, modelling and evaluation in this paper. Table 1 provides the baseline characteristics of the patients. All the 300 patients in the cohort have a history of atrial fibrillation, with 65% having paroxysmal AF, while the others have non-paroxysmal AF (i.e., either persistent or long-standing persistent AF).

**Table 1.** **Baseline characteristics of the patients**.

Baseline Characteristics	Total (N = 300)
Age, years	63.2 (± 10.2)
Men, n (%)	200 (66)
BMI (${\mathrm{kg/m}}^{2}$)	31.1 (± 7.0)
LAV (mL)	135.3 (± 48.4)
EAT Volume (mL)	98.4 (± 47.2)
EAT Attenuation (HU)	–84.5 (± 5.8)
Paroxysmal AF, n (%)	194 (65)

Values are numbers and percentage (%) of the variables (± standard deviation). Abbreviations: AF, atrial fibrillation; BMI, body mass index; EAT, epicardial adipose tissue; HU, Hounsfield Unit; LAV, left atrial volume.

#### 2.1.2 CT Data and Acquisition

CCT was acquired on a 256-slice Multidetector CT scanner (Brilliance iCT, Philips Healthcare, Cleveland, OH, USA). It had a detector collimation of 128 × 0.625 mm with double z-sampling. This scanner had a spatial resolution of 0.625 mm, 0.27 sec gantry rotation time, and temporal resolution of 135 msec. The images were acquired using prospective ECG-gated scanning at 40% of the R-R interval. The CT dataset consisted of 306 consecutive CT scans all of which were deemed to be of adequate image quality for analysis by the readers with the exception of 6 scans which were excluded (2 scans were excluded due to incomplete image acquisition and 4 scans were excluded due to breath hold related artifacts).

The delivery of contrast agent was controlled by automatic bolus tracking with defining a region of interest (ROI) in the center of descending aorta at aortic root level. The initiation of the scan was after a post-threshold delay of 6 sec after the signal attenuation reached a predetermined threshold of 120 HU in the descending aorta. The intravenous contrast administration protocol included 60 mL iohexol (Omnipaque; GE Healthcare; Chicago, IL, USA) at a rate of 5 mL/s with at 120 kV (2% were at 80 kV based on Body Mass Index (BMI) <21 and 9% were at 100 kV based on BMI 21–24). Due to the retrospective nature of the study, we do not have data on the heart rate and rhythm during the scan.

For the reconstruction of image after scanning and evaluation of the quality of images scanned, a dedicated workstation (Extended Brilliance Workspace [EBW] Version V4.5.2.40007, Philips Healthcare, Cleveland, OH, USA) was used. Xres Standard filter (XCB, Philips Healthcare, Cleveland, OH, USA) was used for the purpose of image reconstruction with a reconstruction field of view of 500 mm and image matrix 512 × 512. Raw data reconstruction was obtained by using 0.9 mm slices at 0.45 mm intervals.

#### 2.1.3 Manual Segmentation of EAT and Software Tools

Using the semi-automated post-processing program 3D Slicer (a free, open source software – Version 4.11.0) [44], we analyzed CCT scans and carried out the manual segmentation of EAT using axial views as follows [45]: the EAT was encircled on each 2D slice of the CCT from the bifurcation of the pulmonary artery superiorly to the diaphragm inferiorly, carefully tracing the pericardium to ensure inclusion of epicardial adipose tissue only. This protocol was designed in alignment with the definition of EAT as the fat deposits inside the pericardium (i.e., adipose tissue within the pericardial sac) with the voxels between -190 and -30 Hounsfield units (HU) [8, 46]. 3D Slicer calculates the area of each corresponding EAT section using the HU range of -190 to -30 and estimates EATd and EATv taking into consideration the distance between adjacent planes. The manual segmentation task was performed for all 300 CCTs by physician MSB (with three years of experience in cardiac CT analysis and trained by JDV, a level 3 certified cardiologist with 10 years of experience) and JDV. Both inter-observer correlations (0.822 for EAT volume and 0.934 for EAT attenuation) and intra-observer correlations (0.957 for EAT volume and 0.956 for EAT attenuation) were strong for the manual segmentation task.

All experiments were conducted on a Nvidia Tesla M40 machine with Python programming language using TensorFlow 2.0 Python API machine learning framework (Version 2.7.0, Google Brain, Google Inc., Mountain View, CA, USA) [47].

### 2.2 Image Classification Model

We consider the ResNet (ResNet50) [48] DL architecture for the image classification task of selecting images containing the EAT from the set of all CT images acquired for a given patient, eliminating slices above the bifurcation of the pulmonary trunk or below the cardiac apex. That is, the model classification automatically identifies slices above the superior extent of the left main coronary artery and also those slices below the cardiac apex for elimination in further analysis. A detailed description of the ResNet50 architecture is given in **Supplementary Table 1** and** Supplementary Materials**.

During model training, the input CCT images were resized to 224 × 224 and, as part of the in-training data augmentation, we rotated the images up to ±15° and their intensities normalized. The weights of the models were randomly initialized. Training was carried out for 60 complete epochs using a batch size of 128 images. The binary cross-entropy function was used as the loss function. The Root Mean Squared Propagation (RMSProp) optimizer was chosen as the optimisation technique and set to an initial learning rate of 1e-4 with a step decay schedule.

For our experiments in this study, our 300-patient dataset is divided into two subsets, Dataset 1 (41,979 CT slices) and Dataset 2 (36,428 CT slices), with each subset consisting of 150 patients. We used Dataset 1 for training, validating and evaluating the DL models, and Dataset 2 for evaluating EATv and EATd estimations. Splitting our dataset in this way ensures that in the estimation of EATv and EATd, the DL models trained on Dataset 1 have not seen the CT slices in Dataset 2 during training, allowing us to have a proper evaluation of the proposed methods for EATv and EATd quantification.

The CT image dataset of 150 patients (Dataset 1) of which 23,771 images contain the EAT were used to train, validate and test the ResNet50 model. In particular, 15% of the images were selected; these were then divided into two equal sets representing the validation and test sets. Thus, the number of the training, validation and test (evaluation) images are 35,683, 3148 and 3148, respectively.

Of the 35,683 images in the training set, the number of images with the EAT present and absent are 20,234 and 15449, respectively. We used weighted loss function to address this data imbalance. Let $\left\{\left(x_{1},y_{1}\right),\left(x_{2},y_{2}\right),\ldots ,\left(x_{n},y_{n}\right),\ldots ,\left(x_{N},y_{N}\right)\right\}$, where x is the two-dimensional input image, denote a training set of N samples and $y\in {\left\{0,1\right\}}^{C}$ is a binary one-hot encoded label (i.e., C = 2 in the present case), then the weighted loss function can be written as follows:

(1) Ew(θ)=-1N[λ∑0T0n=1N(xn)ynlog(y^n(xn,θ))+λ1∑T1n=1N(xn)ynlog(y^n(xn,θ))]

where θ denotes the trainable model weights; ${\hat{y}}_{n}\,\left(x_{n},\theta \right)$ is the posterior probability obtained after applying the sigmoid activation function on the model’s output layer; $T_{0}\,\left(x_{n}\right)$ and $T_{1}\,\left(x_{n}\right)$ are functions indicating whether image $x_{i}$ belongs to class 0 or class 1, respectively (i.e., whether the EAT is absent or present in image $x_{i}$, respectively); and $\lambda_{0}$ and $\lambda_{1}$ are weights for penalizing the loss function for false negatives and false positive errors, respectively (i.e., $x_{i}$ that belongs to class 0 is wrongly classified as belonging to class 1 or belongs to class 1 is wrongly classified as belonging to class 0, respectively). The weights, $\lambda_{0}$and $\lambda_{1}$, are given as follows:

(2) $$\lambda_{i}=\frac{1}{k_{i}}\cdot \frac{N}{C}$$

where $k_{i}$ denotes the number of images in class i. In our case, N=35952; class 0 and class 1 are subgroups indicating the collection of samples where EAT is absent and present, respectively; then, $\lambda_{0}=\left(1/20234\right)\times \left(35683/2\right)=0.882$ and $\lambda_{1}=\left(1/15449\right)\times \left(35683/2\right)=1.155$. That is, the images contain the EAT (class 1) are weighted as being more significant than those without the EAT (class 0).

### 2.3 Image Segmentation Model

The image segmentation task of masking the regions of the EAT from the CT slices is performed by the UNet DL model [49]. A detailed description of the UNet architecture is given in **Supplementary Table 2** and **Supplementary Materials**. Briefly, the architecture includes batch normalization to enhance robustness of the model [50] and ‘dropout’ operation [51] to avoid problems associated with model overfitting.

During model training, the input CCT images were resized to 224 × 224 and image rotation, intensity normalization and cropping were performed as part of the in-training data augmentation. The model parameters were randomly initialized, and training proceeded for 60 epochs using a batch size of 64 images. The sparse categorical cross-entropy function and the Adam optimizer were used as the loss function and the optimisation method, respectively, with an initial learning rate of 1e-4 decreasing exponentially at a rate of –0.05 after the first 5 epochs.

For N samples of the training set $\left\{\left(x_{1},y_{1}\right),\left(x_{2},y_{2}\right),\ldots ,\left(x_{n},y_{n}\right),\ldots ,\left(x_{N},y_{N}\right)\right\}$ where x is the two-dimensional input image and y is the two-dimensional segmentation mask, the sparse categorical cross-entropy loss function can be written as follows:

(3) $$E_{w}\,\left(\theta \right)=-\frac{1}{N}\,\left[\sum_{n=1}^{N}y_{n}\,\log\left({\hat{y}}_{n}\,\left(x_{n},\theta \right)\right)\right]$$

where θ denotes the trainable parameters of the model and ${\hat{y}}_{n}\,\left(x_{n},\theta \right)$ is the posterior probability obtained following ‘sigmoid’ activation function on the output layer of the model.

The CT image dataset of 150 patients in Dataset 1 with the EAT present (23,771) were used to train, validate and test the EAT segmentation models. In particular, 15% of these images were selected and divided into two equal sets namely, the validation and test sets. Thus, the numbers of images in training, validation and test sets are 20,207, 1782 and 1782, respectively.

The dice score, or dice similarity coefficient (DSC), is a measure of similarity between the label and predicted segmentation masks. DSC score can be written as follows:

(4) $$D\,S\,C=\frac{2\,\left|A\cap B\right|}{\left(\left|A\right|+\left|B\right|\right)}$$

where A and B represent the two sets (images), |A| and |B| represent the cardinalities (i.e., the number of elements) of set A and B, respectively. The DSC is a useful measure of spatial overlap often used in image segmentation to quantify the accuracy of the predicted mask with respect to the ground truth mask. It is also used as a statistical validation metric by computing the DSC of several images obtained using images from evaluation subset and computing the mean DSC.

### 2.4 Volume Quantification

The estimation of EATv involves the integration (summation) of the interslice volumes. Each interslice volume is approximated by computing the volume between two consecutive slices using the following equation:

(5) $$V=\sum_{i}^{N}v_{i}$$

where V is the estimated EATv and N is the number of slices. $v_{i}$ (the ith interslice volume, i.e., $v_{i}$ the arithmetic mean of the areas of two consecutive slices multiplied by the distance between them in the direction of the z-axis) is computed as follows:

(6) $$v_{i}=\frac{A_{i}+A_{i+1}}{2}\,z_{k_{i}}$$

$z_{k_{i}}$ is the distance between two consecutive slices in the z direction and can be expressed as $z_{k_{i}}=z_{i}\,\hat{k}$, where $\hat{k}$ is the unit vector in the z direction. Also, $z_{i}$ is the perpendicular distance between two consecutive parallel slices; $A_{i}$ and $A_{i+1}$ represent the areas of the two consecutive slices, and $A_{i}$ is defined as follows:

(7) $$A_{i}=n_{i}\,s_{x}\,s_{y}$$

where $n_{i}$ is the number of pixels that constitutes the EAT area on the ith slice; $s_{x}$ and $s_{y}$ are the pixel dimensions in the x and y directions, respectively. If $s=s_{x}=s_{y}$, then $A_{i}=n_{i}\,s^{2}$.

### 2.5 Attenuation Quantification

EATd is estimated by computing the mean attenuation of EAT across all the slices. The intensity values of the EAT for each slice, with range [–190, –30], is extracted using its segmentation mask obtained from the EAT segmentation model to mark the ROI. EATd can be expressed as follows:

(8) $$E\,A\,T\,d=\frac{1}{M}\,\sum_{i=1}^{N\,s}\sum_{j=1}^{N\,p_{i}}x_{i}\,\left(j\right)$$

where $x_{i}\,\left(j\right)$ is the intensity value of pixel j in the ROI of slice i; $N\,p_{i}$ is the number of pixels in the ROI of slice i; N⁢s is the number of slices; and M is the total number of pixels for the of the totality of EATv and can be expressed as:

(9) $$M=\sum_{i=1}^{N\,s}N\,p_{i}$$

Since the size of the input image and output segmentation mask of the segmentation model is 224 × 224, the output segmentation mask was resized to the size of the CT image (512 × 512) using area interpolation (which, in this case of image enlargement, is a bilinear interpolation image processing technique involving resampling using pixel area relation [52]) before extracting the intensity values of the pixels within the ROI on the CT image.

## 3. Results

### 3.1 Image Classification and Image Segmentation Models

**Fig. 2.**
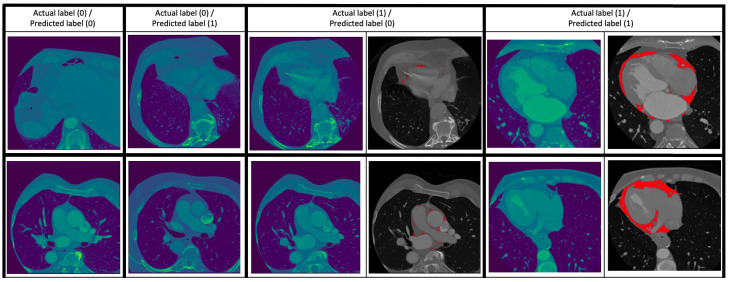
**Some examples of the prediction of the classification model**. Two examples are given for each of the cases (0 – absence of EAT, 1 – presence of EAT): (0/0) (i.e., ground truth/prediction), (0/1), (1/0) and (1/1). For the (1/0) and (1/1) cases, the images to the right show the EAT in red as given by an expert human reader. The (0/1) and (1/0) cases are images which the classification model got wrong.

The performance metrics of the ResNet50 classification model on the evaluation dataset of N = 3148 are precision (0.980), recall (0.986) and $F_{1}$ score (0.983) and the confusion matrix is given in Table 2. Some examples of the predictions of the classification model are given in Fig. 2.

**Table 2.** **Confusion matrices for the ResNet50 classification model using the evaluation dataset (N = 3148) with class 0 (absence of the EAT in an image) and class 1 (presence of the EAT in an image)**.

	Predicted Label (0)	Predicted Label (1)
Actual Label (0)	1388	24
Actual Label (1)	34	1702

**Fig. 3.**
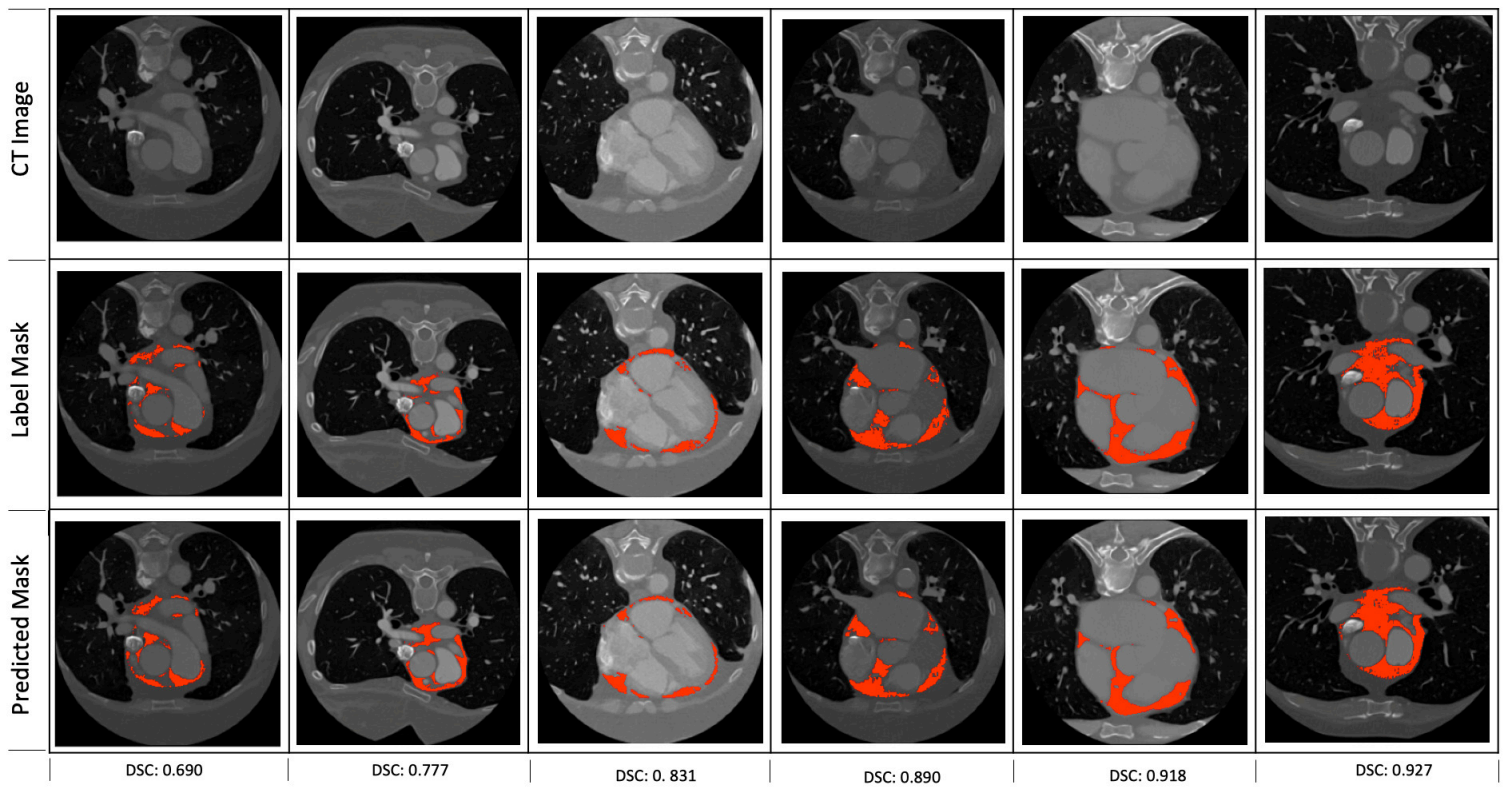
**Some examples of the predictions of the segmentation model**. The corresponding dice scores are shown at the bottom of each of the examples.

The performance metrics of the segmentation model on the 1782 evaluation set are given as follows: the mean DSC is 0.844 (± 0.19 standard deviation); 25%, 50% (median) and 75% percentiles of DSC are 0.81, 0.84 and 0.87, respectively; the maximum DSC is 0.95. To ensure that the proposed segmentation model is robust, the mean (± std.), maximum and median DSC of this model (0.844 (± 0.19), 0.95 and 0.84, respectively) is compared with the results from a 5-fold cross-validation of the same model architecture are presented in Table 3 (i.e., 20% of Dataset 1 is used as subsamples/validation set and each of the 5 subsamples used exactly once, creating 5 models); the best of the five models (model 4) gives the mean (± std.), maximum and median DSC as 0.851 (± 0.17), 0.96, and 0.84, respectively. Fig. 3 gives some examples of the predictions of the segmentation model with varying DSC.

**Table 3.** **Comparing the mean, maximum and median Dice Scores of five UNet segmentation models calculated from 5-fold cross-validation**.

	Mean (std. dev.)	Max.	Median
Model 1	0.831 (± 0.14)	0.91	0.83
Model 2	0.826 (± 0.19)	0.92	0.84
Model 3	0.818 (± 0.21)	0.94	0.82
Model 4	0.851 (± 0.17)	0.96	0.84
Model 5	0.838 (± 0.22)	0.91	0.82

### 3.2 Volume and Attenuation Quantification

**Fig. 4.**
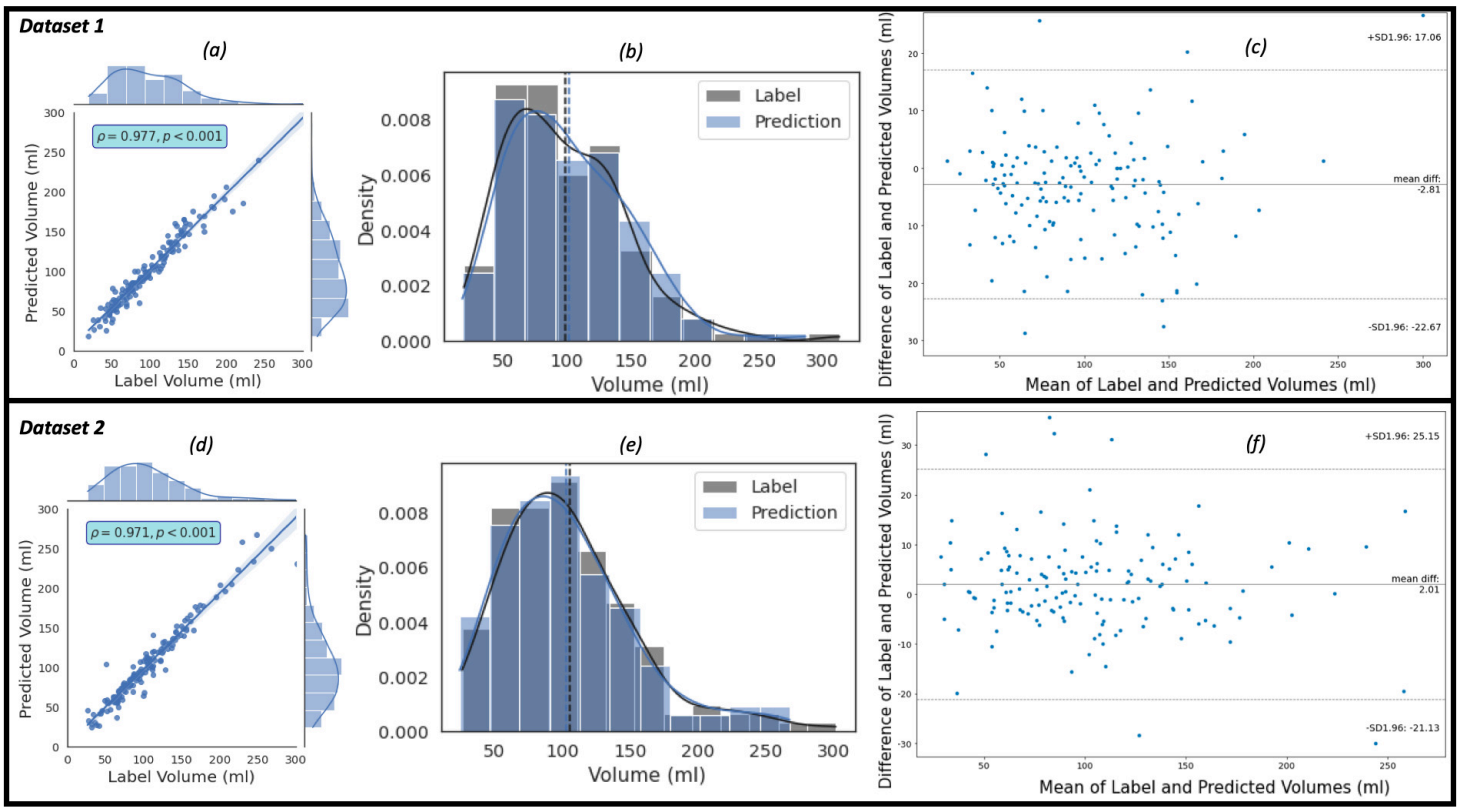
**The plots of the predicted volume against the label volume**. Plot (a) represents the regression plot; plot (b) represents the kernel density estimates and histogram plots of the two variables (label volume and predicted volume) with the dashed vertical lines representing the arithmetic mean of the distributions. The symbols ρ and p represent the *p*-value and Pearson correlation coefficient, respectively. Plot (c) represents the Bland-Altman plot of the predicted volumes against the label volumes where the lower and upper dashed horizontal lines are the confidence interval at 95%. (a–c) show the plots for Dataset 1. For Dataset 2, plot (d) represents the regression plot; plot (e) represents the kernel density estimates and histogram plots of the two variables (label volume and predicted volume); plot (f) represents the Bland-Altman plot of the predicted volumes against the label volumes.

The regression, the kernel density estimates and the Bland-Altman plots of the predicted volumes against the label volumes computed for the study population of Dataset 1 (the ‘training’ dataset) and Dataset 2 (the ‘evaluation’ dataset), each of which consists of 150 patients, using the proposed framework are shown in Fig. 4. Fig. 4a,c show the regression plots with volume estimated using Eqns. 5,6,7. In our case, $s_{x}=s_{y}$ and $z_{k_{i}}$ is approximately between 2.2 mm and 4.5 mm. The Pearson correlation coefficient, ρ, between the label and predicted EATv are 0.977 ($R^{2}$ = 0.954) and 0.971 ($R^{2}$ = 0.943) for the populations in Dataset 1 and Dataset 2, respectively. The arithmetic means (93.36 mL for label and 101.16 mL for prediction in Dataset 1, and 105.30 mL for label and 103.29 mL for prediction in Dataset 2) of the distributions (the dashed vertical lines on the kernel density estimates plot of Fig. 4b,e) show close agreements between the label and prediction of the mean EATv of the populations. Bland-Altman plots Fig. 4c,f indicates 95% of the difference between the label and predicted EATv values will be between –22.67 mL and +17.06 mL of the mean EATv difference value of –2.81 mL for Dataset 1, and between –21.13 mL and +25.15 mL of the mean EATv difference value of +2.01 mL for Dataset 2.

**Fig. 5.**
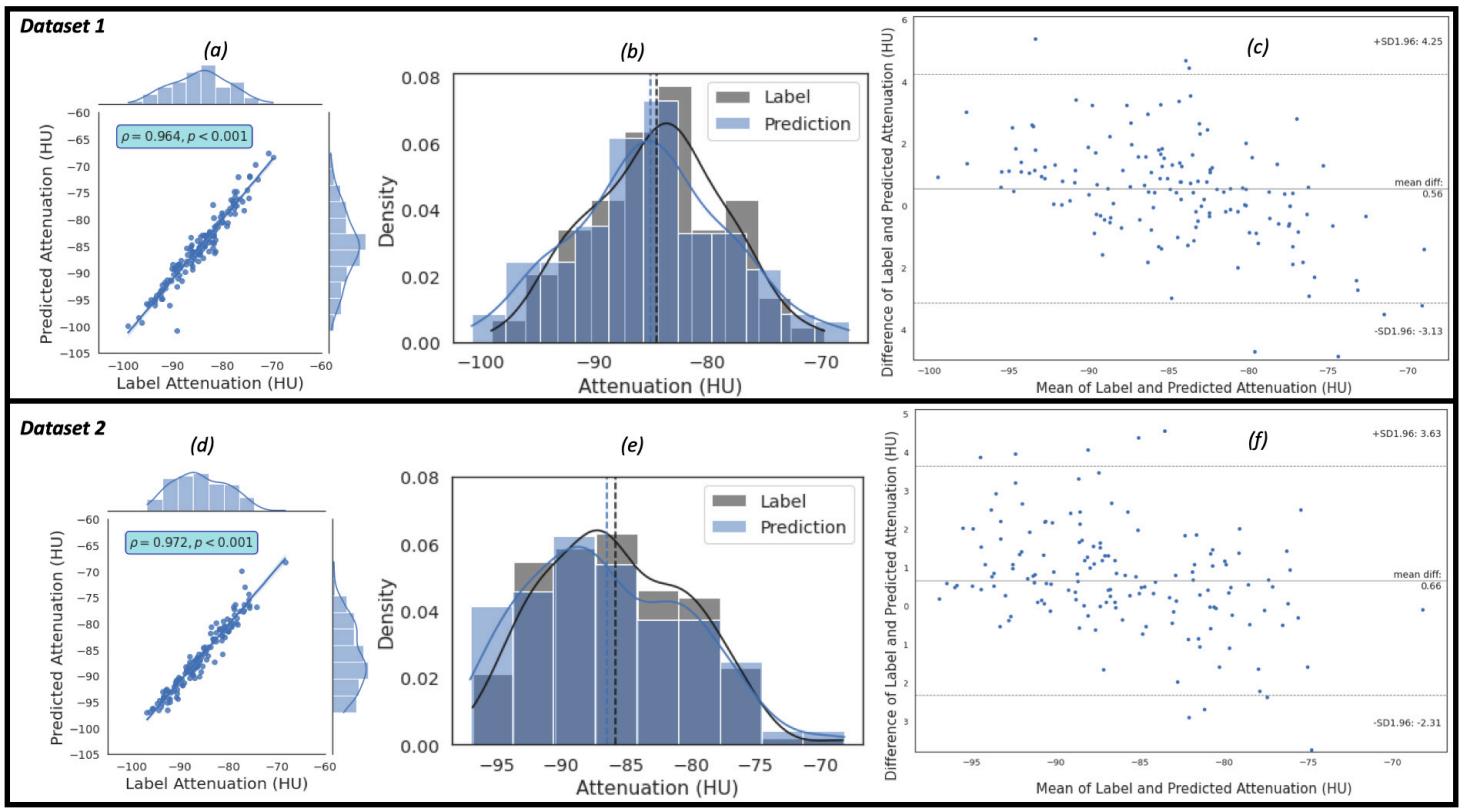
**The plots of the predicted attenuation against the label attenuation**. Plot (a) represents the regression plot and plot (b) represents the kernel density estimates and histogram plots of the two variables (label and predicted mean attenuations) with the dashed vertical lines representing the arithmetic mean of the distributions. The symbols ρ and p represent the *p*-value and Pearson correlation coefficient, respectively. Plot (c) represents the Bland-Altman plot of the predicted versus the label mean attenuations where the lower and upper dashed horizontal lines are the confidence interval at 95%. (a–c) show the plots for Dataset 1. For Dataset 2, plot (d) represents the regression plot; plot (e) represents the kernel density estimates and histogram plots of the two variables (label volume and predicted volume); plot (f) represents the Bland-Altman plot of the predicted volumes against the label volumes.

Similarly, the regression, the kernel density estimates and the Bland-Altman plots of the predicted EATd versus the label EATd estimated for the study population of Dataset 1 and Dataset 2 are given in Fig. 5. Fig. 5a,c show the regression plots, and the Pearson correlation coefficient between the label and predicted EAT mean attenuation are 0.964 ($R^{2}$ = 0.930) and 0.972 ($R^{2}$ = 0.945) for the populations in Dataset 1 and Dataset 2, respectively. The arithmetic means (–84.51 HU for label and –85.07 HU for prediction in Dataset 1, and –85.78 HU for label and –86.44 HU for prediction in Dataset 2) of the distributions (in Fig. 5b,e) show close agreements between the label and prediction of the mean attenuation of the populations. Bland-Altman plots Fig. 5c,f) indicates 95% of the difference between the label and predicted EATd values will be between –3.13 HU and +4.25 HU of the mean EATd difference value of 0.56 HU for Dataset 1, and between –2.31 HU and +3.36 HU of the mean EATd difference value of 0.66 HU for Dataset 2. Moreover, the estimation of EATv and EATd took approximately 39.54 sec and 30.74 sec per patient, respectively. Some examples of the label (ground truth) EATv and EATd values and the predicted values from the proposed framework are given in **Supplementary Table 3**.

## 4. Discussion

In our task of quantifying EATv and EATd, we developed the ResNet50 DL classification model for selecting images containing the EAT from the set of CT images acquired for a given patient. We then used a UNet segmentation model for obtaining the segmentation masks marking the regions of the EAT from the selected images. The estimation of EATv and EATd are computed using the segmentation masks and by extracting the intensity values of the EAT from the selected CT images. Using the regression, the kernel density estimates and the Bland-Altman plots, we have shown that the proposed framework is able to estimate EATv and EATd with a high degree of accuracy. To summarize, we reported the performance of the classification model in terms of precision, recall and $F_{1}$ score as 0.980, 0.986 and 0.983, respectively, and of the segmentation model in terms of mean and median DSC as 0.844 (± 0.19) and 0.84, respectively. Using the evaluation set, Dataset 2 (36,428 CT slices from 150 subjects), in which neither the classification model nor the segmentation model has been exposed to any of its slices during training, our results showed a Pearson correlation coefficient of 0.971 ($R^{2}$ = 0.943) between the label and predicted EATv with the 95% limits of agreement range from Bland-Altman plot being –21.13 mL and +25.15 mL and the mean EATv difference value being +2.01 mL. For EATd estimation on Dataset 2 evaluation set, our results showed a Pearson correlation coefficient of 0.972 ($R^{2}$ = 0.945) between the label and predicted EATd with the 95% limits of agreement range from Bland-Altman plot being –2.31 HU and +3.36 HU and the mean EATd difference value being 0.66 HU. These results in the analysis of EATd strengthens the correctness of the results obtained using the approaches proposed in this paper for EAT segmentation and EATv estimation. In summary, this work contributes to the field of DL applications in medical imaging by proposing a robust and fast fully automated framework for EATv and EATd estimation with a high degree of accuracy that can be used at patient-level in hospitals or for projects requiring quantification of EATv and EATd for epidemiological scale studies and analysis.

### 4.1 Comparison with Existing Work

The performance of the method proposed in [39] was evaluated for EAT segmentation on 10% of the dataset of 250 subjects (i.e., 25 in the evaluation set) and gave a median DSC of 0.823. EATv on the 250 patients gave a correlation of ($R^{2}$ <0.924). The 95% limits of agreement ranged from Bland-Altman plots is approximately –27 mL to 23 mL. The approach presented in this paper significantly differs with the method proposed in [39] in that, unlike in [39] where the task of quantifying EAT is divided into 3 separate tasks (namely, slice selection task, heart localization task, and pericardium line detection) and a further post-processing of the outcome via thresholding, our approach only involved two tasks: slice selection and EAT segmentation. To emphasize, our approach does not require any further post-processing. We note that the goal of the slice selection task in our method and the method in [39] are the same except that we have used a more recent classification architecture (i.e., ResNet50 [48]). Moreover, the authors of [39] have extended their approach to a larger multi-centre cohort in [41] and have reported a median DSC of 0.873 for EAT segmentation on 10% of 614 CT dataset and EATv evaluation of ($R^{2}$ <0.974) on a dataset of 614 studies. The 95% limits of agreement ranged from Bland-Altman plots is –19.59 mL to 21.42 mL. A further extension of the work in [39] is given [42].

Also, the method proposed in [40] was trained on a dataset of 88 subjects, and reported a mean DSC score of 0.973 using an evaluation dataset of only 15 subjects; thus, the evaluation dataset is not large enough for making a fair comparison with other methods. In addition, our approach differs from the method presented in [40] in that it does not require smoothing operation by solving a differential equation nor any post-processing step via thresholding.

### 4.2 Limitation and Future Work

Our models were trained on a dataset from a single hospital system. The data augmentation techniques, the batch normalization and dropout operations and the weighted loss function for addressing data imbalance used during model training may have improved the chance that performance may not deteriorate significantly from datasets from elsewhere but it would be useful assessing the performance of the models using an external dataset. If needed, the performance of the models may then be improved with training on multi-centre datasets to enhance generalizability with little or no modification to the proposed methods. Approaches that may be explored to address model generalization issues include transfer learning and federated-learning [53].

In relation to gender, men constitute the majority (66%) of the dataset we have used for model training. In the evaluation of our models trained with this dataset using 5-fold cross-validation (Table 3), there is no indication of biasness of the models at slice level towards a particular gender. Biases in the training datasets can affect performance of DL models. As such, future work on this research would focus on investigating the estimation of EATv and EATd at patient-level for biasness. A possible approach for addressing biasness in CT images is by balancing the dataset using generative models in the form of data augmentation. An in-depth discussion on generative models is beyond the scope of this paper and we refer readers to [54] for more details on this technique.

Our framework focused on EATv and EATd quantification for CT images, future work will focus on training using heterogenous multi-centre dataset as well as on analysis of EAT for cardiovascular risk and outcome prediction. Future direction of this work will also include using machine learning methods for quantifying the distribution of EAT given that the location of EAT is a disease-specific risk factor (e.g., thickness of peri-atrial EAT being a predictor of AF recurrence [55]).

## 5. Conclusions

We proposed a novel and clinically useful framework that consists of DL models for EAT quantification. The framework provides a fast and robust strategy for accurate EAT segmentation, and volume (EATv) and attenuation (EATd) quantification tasks. It fully automates the process of computing EATv and EATd. The framework we have proposed in this paper will be useful to clinicians and other practitioners as a first step which they can build upon in order to develop DL models for carrying out reproducible EAT quantification at patient level or for large cohorts and high-throughput projects, creating prognostic EAT data for better further analyses.

## Data Availability

The datasets presented in this article are not publicly available because restrictions apply to the availability of these raw data, which were used under license for the current study from Georgetown University Institutional Review Board. Generated anonymized dataset are however available from the authors upon reasonable request and with permission of Georgetown University Institutional Review Board. Requests to access the datasets should be directed to Jose D. Vargas, jose.vargas@nih.gov.

## Abbreviations

AF, atrial fibrillation; CCT, cardiac CT; CT, computed tomography; DL, deep learning; DSC, dice similarity coefficient; EAT, epicardial adipose tissue; EATv, EAT volume; EATd, EAT density (mean EAT attenuation).

## Supplementary Material

Supplementary material associated with this article can be found, in the online version, at https://doi.org/10.31083/j.rcm2312412.
