# Supplementary material for: Quantification of Epicardial Adipose Tissue Volume and Attenuation for Cardiac CT Scans Using Deep Learning in a Single Multi-Task Framework
Source: Rev Cardiovasc Med. 2022 Dec 20;23(12):412. doi: 10.31083/j.rcm2312412 (PMC11270472; doi:10.31083/j.rcm2312412)
Supplement: Supplementary file 1 [file 2153-8174-23-12-412-s1.docx]

**Quantification of Epicardial Adipose Tissue Volume and Attenuation for Cardiac CT Scans using Deep Learning in a Single Multi-task Framework**

**Supplementary Materials**

**1. Image Classification Model**

The ResNet50 architecture given in Supplementary Table 1 consists of five stages. The first stage consists of one convolutional layer while each of the remaining four stages consists of several blocks of convolutional layers with skip connections. These blocks include identity blocks (i.e. contain no convolutional layer in the skip connection path) and convolutional blocks (i.e. contain convolutional layer in the skip connection path). The last convolutional layer of the architecture is followed by a global average pooling operation and a dense layer of 1 unit (neuron) with the sigmoid nonlinear activation function applied. The output of the model yields a binary prediction (i.e. an image is with/without the EAT). The model has a total of 23,583,489 parameters out of which 23,530,369 are trainable.

**Supplementary Table 1. Configuration of the ResNet50 DL Architecture. The convolutional layer parameters are denoted as “conv2-(number of filters)”, where “conv2” denotes 2D convolution operation and the height and width of the 2D convolution window is 2×2. Each of the convolutional or identity blocks is followed by the number of filters** $\boldsymbol{n}_{\boldsymbol{i}}$ **of its three 2D convolutional operations as in “convolutional block** $\boldsymbol{x}$ **(**$\boldsymbol{n}_{\boldsymbol{1}}$**,** $\boldsymbol{n}_{\boldsymbol{2}}$**,** $\boldsymbol{n}_{\boldsymbol{3}}$**)”. Two or more identity blocks stacked together are denoted as “[identity block** $\boldsymbol{x}$ **(**$\boldsymbol{n}_{\boldsymbol{1}}$**,** $\boldsymbol{n}_{\boldsymbol{2}}$**,** $\boldsymbol{n}_{\boldsymbol{3}}$**)] ×** $\boldsymbol{k}$**”, where** $\boldsymbol{k}$ **denotes the number of blocks stacked together.**

| **Stage name** | **Layer/Block Name** | **Output size** |
| --- | --- | --- |
|  | Input | 224 × 224  (one channel image) |
| Stage 1 | zero padding  conv2-64  batch normalization  ReLU  zero padding  maxpool (kernel size = 2, stride = 2) | 230 × 230  112 × 112  112 × 112  112 × 112  114 × 114  56 × 56 |
| Stage 2 | convolutional block 1 (64, 64, 256)  [identity block 1 (64, 64, 256)] × 2 | 56 × 56  56 × 56 |
| Stage 3 | convolutional block 2 (128, 128, 512)  [identity block 2 (128, 128, 512)] × 3 | 28 × 28  28 × 28 |
| Stage 4 | convolutional block 3 (256, 256, 1024)  [identity block 3 (256, 256, 1024)] × 5 | 14 × 14  14 × 14 |
| Stage 5 | convolutional block 4 (512, 512, 2048)  [identity block 4 (512, 512, 2048)] × 2 | 7 × 7  7 × 7 |
|  | global average pooling | 2048 |
|  | fully connected layer (1 unit, sigmoid activation function) | 1 |

**2. Image Segmentation Model**

The UNet architecture is given in Supplementary Table 2 includes batch normalization following every convolutional layer. ‘Dropout’ operation involving the dropping out 30% hidden neurons is performed on the first six consecutive up-sampling convolutional layers of the architecture. The output of the model is a segmentation mask with the background pixels labelled 0 and the foreground pixels (region of the EAT) labelled l (i.e. the output of the UNet model has 2 channels; thus, in order to create a mask, the prediction for each pixel will be the channel with the highest probability). The model has 29,812,034 parameters out of which 29,800,514 parameters are trainable.

**Supplementary Table 2. Configuration of the UNet DL Architecture. The convolutional layer parameters are denoted as “conv2 (dimension of output, number of filters)” where “conv2” denotes 2D convolution operation. “conv2-T (dimension of output, number of filters)” denotes 2D transpose convolution layer. The conv2 and conv2-T operations have ‘same’ padding (i.e. output and input of the operation have the same height and width).**

| **Layer/Block/Operation Name** | **Layer/Block/Operation Name** |
| --- | --- |
| \| INPUT IMAGE \| \| --- \|   Input Image (224×224, 1) | \| OUTPUT IMAGE \| \| --- \|   Output Image (224×224, 2) |
| \| Block 1 \| \| --- \|   Conv2 (112×112, 64) [kernel size = 3×3, stride = 2]  Leaky ReLU  Conv2 (112×112, 64) [kernel size = 3×3, stride = 1]  Leaky ReLU | \| Block 10 \| \| --- \|   Skip connection (concatenates outputs of Blocks 1 and 9)  Conv2-T (224×224, 2) [kernel size = 3×3, stride = 2]  softmax |
| \| Block 2 \| \| --- \|   Conv2 (56×56, 128) [kernel size = 3×3, stride = 2]  Batch normalization  Leaky ReLU  Conv2 (56×56, 128) [kernel size = 3×3, stride = 1]  Batch normalization  Leaky ReLU | \| Block 9 \| \| --- \|   Skip connection (concatenates outputs of Blocks 2 and 8)  Conv2-T (112×112, 64) [kernel size = 3×3, stride = 2]  Batch normalization  ReLU  Conv2-T (112×112, 64) [kernel size = 3×3, stride = 1]  Batch normalization  ReLU |
| \| Block 3 \| \| --- \|   Conv2 (28×28, 256) [kernel size = 3×3, stride = 2]  Batch normalization  Leaky ReLU  Conv2 (28×28, 256) [kernel size = 3×3, stride = 1]  Batch normalization  Leaky ReLU | \| Block 8 \| \| --- \|   Skip connection (concatenates outputs of Blocks 3 and 7)  Conv2-T (56×56, 128) [kernel size = 3×3, stride = 2]  Batch normalization  Dropout  ReLU  Conv2-T (56×56, 128) [kernel size = 3×3, stride = 1]  Batch normalization  Dropout  ReLU |
| \| Block 4 \| \| --- \|   Conv2 (14×14, 512) [kernel size = 3×3, stride = 2]  Batch normalization  Leaky ReLU  Conv2 (14×14, 512) [kernel size = 3×3, stride = 1]  Batch normalization  Leaky ReLU | \| Block 7 \| \| --- \|   Skip connection (concatenates outputs of Blocks 4 and 6)  Conv2-T (28×28, 256) [kernel size = 3×3, stride = 2]  Batch normalization  Dropout  ReLU  Conv2-T (28×28, 256) [kernel size = 3×3, stride = 1]  Batch normalization  Dropout  ReLU |
| \| Block 5 \| \| --- \|   Conv2 (7×7, 1024) [kernel size = 3×3, stride = 2]  Batch normalization  Leaky ReLU  Conv2 (7×7, 1024) [kernel size = 3×3, stride = 1]  Batch normalization  Leaky ReLU | \| Block 6 \| \| --- \|   Conv2-T (14×14, 512) [kernel size = 3×3, stride = 2]  Batch normalization  Dropout  ReLU  Conv2-T (14×14, 512) [kernel size = 3×3, stride = 1]  Batch normalization  Dropout  ReLU |

**3. Volume and Attenuation Quantification**

Supplementary Table 3 shows 10 examples of the label (ground truth) EATv and EATd values and the predicted values from the proposed framework.

**Supplementary Table 3. Examples of label and predicted EATv and EATd values for 10 selected patients**

| No. | Label EATv | Predicted EATv | Label EATd | Predicted EATd |
| --- | --- | --- | --- | --- |
| 1 | 52.27 | 59.69 | -79.01 | -76.80 |
| 2 | 195.12 | 189.59 | -85.52 | -86.31 |
| 3 | 151.79 | 149.16 | -88.24 | -87.99 |
| 4 | 105.66 | 111.69 | -92.06 | -93.13 |
| 5 | 60.86 | 60.50 | -84.50 | -85.00 |
| 6 | 161.22 | 158.87 | -90.41 | -90.28 |
| 7 | 90.54 | 89.97 | -80.19 | -81.23 |
| 8 | 73.74 | 70.76 | -81.43 | -83.26 |
| 9 | 59.93 | 63.15 | -76.86 | -77.36 |
| 10 | 89.58 | 84.09 | -87.49 | -88.48 |

Abbreviations: EAT, epicardial adipose tissue; EATv, EAT volume; EATd, mean EAT attenuation (EAT density)
